# The role of peptidyl-prolyl isomerase Pin1 in neuronal signaling in epilepsy

**DOI:** 10.3389/fnmol.2022.1006419

**Published:** 2022-10-11

**Authors:** Yuwen Chen, Xiaojun Hou, Jiao Pang, Fan Yang, Angcheng Li, Suijin Lin, Na Lin, Tae Ho Lee, Hekun Liu

**Affiliations:** ^1^Institute of Basic Medicine, The School of Basic Medical Sciences, Fujian Medical University, Fuzhou, China; ^2^Fuzhou Children’s Hospital of Fujian Medical University, Fuzhou, China; ^3^Department of Laboratory Medicine, Xiang’an Hospital of Xiamen University, School of Medicine, Xiamen University, Xiamen, China

**Keywords:** Pin1, neuronal signaling, neurodegeneration, epilepsy, synapses

## Abstract

Epilepsy is a common symptom of many neurological disorders and can lead to neuronal damage that plays a major role in seizure-related disability. The peptidyl-prolyl isomerase Pin1 has wide-ranging influences on the occurrence and development of neurological diseases. It has also been suggested that Pin1 acts on epileptic inhibition, and the molecular mechanism has recently been reported. In this review, we primarily focus on research concerning the mechanisms and functions of Pin1 in neurons. In addition, we highlight the significance and potential applications of Pin1 in neuronal diseases, especially epilepsy. We also discuss the molecular mechanisms by which Pin1 controls synapses, ion channels and neuronal signaling pathways to modulate epileptic susceptibility. Since neurotransmitters and some neuronal signaling pathways, such as Notch1 and PI3K/Akt, are vital to the nervous system, the role of Pin1 in epilepsy is discussed in the context of the CaMKII-AMPA receptor axis, PSD-95-NMDA receptor axis, NL2/gephyrin-GABA receptor signaling, and Notch1 and PI3K/Akt pathways. The effect of Pin1 on the progression of epilepsy in animal models is discussed as well. This information will lead to a better understanding of Pin1 signaling pathways in epilepsy and may facilitate development of new therapeutic strategies.

## Introduction

Epilepsy is a common, chronic, and severe neurological disorder. Recurrent and unpredictable interruptions of normal brain function are the characteristic symptoms of epileptic seizures (Fisher et al., 2005; Moshé et al., 2015), which result from transient abnormal synchronization of neurons in the brain (Moshé et al., 2015). Many studies have demonstrated electrophysiological abnormalities in epilepsy. High-frequency oscillations (HFOs), repetitive HFOs and spikes (rHFOSs) and sleep spindles are typical electrophysiological biomarkers of epileptogenesis (Perucca et al., 2019). Neurotransmitters and neuroendocrine axes are critical in the onset of epilepsy (Camfield et al., 2017). Although the molecular mechanism of epilepsy has not been fully elucidated, there is growing evidence for synaptic transmission in the pathological process of epilepsy (Amador et al., 2020; Puhahn-Schmeiser et al., 2021), and altered dynamics of inhibitory synapses and excitatory synapses may be a critical signature of epileptic networks (Vannini et al., 2020).

The peptidyl prolyl *cis-trans* isomerase (PPIase) superfamily consists of four families characterized by their structural differences: cyclophilins, FK506-binding proteins (FKBPs), parvulins, and the protein phosphatase (PPase) 2A phosphatase activator (PTPA) (Li et al., 2021). Discovered in 1996 as a substrate of NIMA (never in mitosis, gene A), PPIase NIMA-interacting 1 (Pin1) protein is associated with mitosis (Lu et al., 1996; Fagiani et al., 2021). It consists of 163 amino acid residues with a relative molecular mass of 18 kDa and contains 1 nuclear localization signal and 2 functional domains (Li et al., 2021). Pin1-catalyzed isomerization, which is detected by cis and trans proline isomer-specific antibodies (Nakamura et al., 2012a; Kondo et al., 2015), has the ability to function as a molecular timer to synchronize cellular processes (Lu et al., 2007; Liou et al., 2011; Zhou and Lu, 2016).

Recently, emerging evidence has demonstrated that Pin1 is closely related to many cellular processes, such as the cell cycle, cell proliferation, cell motility, and apoptosis, by its isomerization phosphorylated substrates and postphosphorylation regulation (Ranganathan et al., 1997; Hilton et al., 2015; Lin et al., 2015; Feng et al., 2017; Risal et al., 2017). In addition, Pin1 also exerts a pivotal effect on multiple physiological processes, including the immune response, neuronal differentiation, and tumorigenesis (Sacktor, 2010; Tun-Kyi et al., 2011; Daza-Martin et al., 2019). Pin1 activity is tightly regulated through finely tuned cellular pathways downstream of phosphorylation signaling, and its dysregulation under pathological conditions is related to multiple diseases (Fagiani et al., 2021; Li et al., 2021). In particular, Pin1 regulates several neuronal proteins, such as Tau, amyloid precursor protein (APP), and α-synuclein (Li et al., 2021) and thus has a significant impact on the development of many neurodegenerative diseases.

Spontaneous seizures have been observed in Pin1-knockout mice without any induction, suggesting that Pin1 may be a neuroprotective gene in the development of epilepsy, and its function may be associated with the regulation of excitatory and inhibitory synapses (Antonelli et al., 2014, 2016; Tang et al., 2017). Specifically, Pin1 may be associated with excitatory glutamate receptors N-methyl-D-aspartic acid [(NMDA) receptors and alpha-amino-3-hydroxy-5-methyl-4-isoxazolepropionic acid (AMPA) receptors] as well as inhibitory neurotransmitter receptors gamma-aminobutyric acid [(GABA) receptors and glycine receptors], which can effectively regulate the release of neurotransmitters and are significant in the progression of epilepsy. Electrophysiological studies of ion channels have also revealed some of the pathophysiological mechanisms underlying Pin1 and epilepsy (Antonelli et al., 2014, 2016; Hu J. H. et al., 2020; Hou et al., 2021).

Given the critical role of Pin1 in neurological diseases, we discuss in this review the recent findings of the dysregulation, mechanisms, and biological functions of Pin1 in epilepsy.

## The structure and function of Pin1

Pin1 was first found to be an essential PPIase that regulates mitosis, presumably by interacting with NIMA and attenuating its mitosis-promoting activity (Lu et al., 1996). Pin1 can specifically catalyze the isomerization of phosphorylated proline-directed serine or threonine (pS/T-P) (Wang et al., 2020; Li et al., 2021). The N-terminal WW domain (residues 1–39) and C-terminal PPIase domain (residues 50–163) are two main protein functional domains of Pin1. The WW domain performs the function of recognition and binding to the pSer/Thr-Pro motif of the substrate, while the PPIase domain is responsible for *cis-trans* isomerization and catalytic activity (Lu et al., 1999; Wang et al., 2020; Fagiani et al., 2021; Li et al., 2021). Since the WW domain specifically interacts with pSer-Pro or pThr-Pro motifs, which are the key phosphorylation sites of the substrates, Pin1 regulates diverse cellular processes in cell signaling pathways, such as cell cycle progression, cellular stress responses, development, neuronal function, immune responses, and cell death (Zhou et al., 2000; Lu and Zhou, 2007; Wang et al., 2020). Notably, Pin1 is expressed at very high levels in neurons (Nakamura et al., 2012b). Moreover, Pin1 function is inhibited in human central nervous system (CNS) disorders, including Alzheimer’s disease, Parkinson’s disease and schizophrenia, by multiple mechanisms (Driver et al., 2015; Fagiani et al., 2021), which supports the idea that it is an essential protective gene in the nervous system. The selected Pin1 substrates associated with the nervous system are summarized in Table 1.

**TABLE 1:** Selected Pin1 substrates.

Protein	Substrate regulation by Pin1	Pin1-binding motif	Cellular consequence of Pin1 substrates activation	References
PSD95	Structural rearrangements	T287/S290/S295	Negatively affecting its ability to interact with NMDARs	Antonelli et al., 2016
NMDAR	Altered NMDARs surface trafficking	NR2A and NR2B	Regulating hyper-excitability	Tang et al., 2017
CaMKII	Conformational change, auto-phosphorylation of Thr286.	T176	Activating CaMKII, increasing expression of total AMPA receptors and phosphorylating GluA1 Ser831	Hou et al., 2021
PKC	Phosphorylation Thr of the turn motif, converted PKC isozymes	The hydrophobic motif of conventional PKC isozymes	Dephosphorylating and ubiquitination PKC	Abrahamsen et al., 2012; Yang and Igumenova, 2013
PKA	Phosphorylation in the WW domain of Pin1	S16	Abolishing its binding capacity to a phosphorylated Tau peptide	Smet-Nocca et al., 2013
Neuroligin2	Phosphorylation	S714	Negatively modulating gephyrin–NL2 interaction	Antonelli et al., 2014

## Pin1 and epilepsy

### Pin1 has protective effects against the pathology of epilepsy

Although many signaling pathways have been reported to be involved in the pathology of epilepsy by participating in seizure-induced cognitive dysfunction or neuronal apoptosis (Cendes et al., 2005; Henshall and Engel, 2013), the exact molecular mechanism of epilepsy is still unclear. In addition, Pin1 is involved in many cellular processes because of its plentiful protein targets (Liou et al., 2011; Yuan et al., 2011; Tang et al., 2017). Pin1 interacts with many neuronal proteins, and its deficiency is implicated in neurodegeneration (Balastik et al., 2007; Driver and Lu, 2010; Rudrabhatla and Pant, 2010; Tang et al., 2017). Pin1 can inhibit the protein synthesis required to sustain the late phase of long-term potentiation (LTP) (Keeney et al., 2012; Tang et al., 2017). In addition, Pin1 regulates conformational changes in the neuronal protein gephyrin, which is involved in the development of temporal lobe epilepsy (TLE) (Fang et al., 2011), and it modulates glycine receptor function as well (Zita et al., 2007). Therefore, it is reasonable to hypothesize that Pin1 is associated with the development of epilepsy.

Pin1-deficient mice have significantly upregulated seizure susceptibility in chemically induced models and develop age-dependent spontaneous epilepsy without any induction, strongly suggesting that Pin1 may be an important neuroprotective gene and that deletion of Pin1 may contribute to the occurrence of epilepsy (Hou et al., 2021). Recent studies have shown that the expression of Pin1 is remarkably downregulated in epileptic patients as well as experimental epileptic mouse models (Tang et al., 2017). The expression of Pin1 is dramatically decreased in the neocortex of epilepsy patients. Additionally, a pilocarpine-induced epileptic mouse model has also been used to verify the expression of Pin1 in epilepsy, and the results are consistent with those in humans (Tang et al., 2017), which supports the antiepileptic effect of Pin1. This study provided direct evidence that Pin1 is essential in regulating epileptic seizures.

### Pin1 regulates the synapses and ion channels in neurons

**FIGURE 1:**
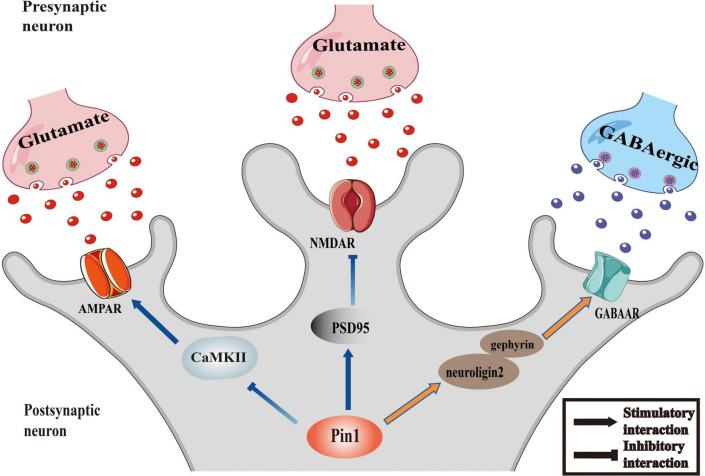
Pin1 as regulator of synaptic activity. The function of AMPAR could be inhibited by the interaction between Pin1 and CaMKII. Pin1 could down regulate the NMDA-mediated synaptic transmission by binding to PSD95. And the transmission of GABAergic will be modulated by Pin1 interfering the function of neuroligin2-gephyrin complex.

The imbalance of excitability and inhibition of a large number of neurons is a significant cause of epileptic seizures. Inhibitory and excitatory neurotransmitters are critical regulators of hyperexcitability in epilepsy (Van Liefferinge et al., 2013). Abnormal increases in excitability, inadequate inhibitory mechanisms, or a combination of both lead to the excitability of the neurons in the epileptic foci, resulting in the uncontrolled spontaneous abnormal synchronous discharge of these neurons, followed by repeated seizures (Moshé et al., 2015; Camfield et al., 2017). Among them, excitatory glutamate receptor NMDA receptors, AMPA receptors and inhibitory neurotransmitter receptor GABA receptors, which are all regulated by Pin1, are involved in the regulation of epileptic activity (He et al., 2011; Rogawski, 2013; Lippman-Bell et al., 2016). The proposed relationship between Pin1 and synapses is presented as a conceptual model in Figure 1.

#### N-methyl-D-aspartic acid receptors

Ionotropic glutamate NMDA receptors are important regulators of glutamatergic synaptic transmission in the CNS and modulate the maturation and plasticity of glutamate synapses (Ladépêche et al., 2014). Recent studies have suggested the possible role of lateral diffusion in the surface distribution of NMDA receptors, which provides a powerful way to rapidly affect the content and composition of synaptic receptors (Peng et al., 2010; Ladépêche et al., 2014). The increased number of cell surface NMDARs is responsible for cell injury and neuronal death during epileptogenesis, and therapies that directly antagonize NMDA receptors may prevent excitotoxicity, which develops after various pro-epileptogenic brain injuries (Frasca et al., 2011; Naylor et al., 2013; Tang et al., 2017).

The relationship between Pin1 and NMDA receptors has recently been recognized. PSD-95 is a membrane-associated guanylate kinase with a catalytically inactive guanylate kinase domain, a Src homology 3 (SH3) domain and three PDZ (PSD-95/Disks large/zona occludens-1) domains, which allow it to anchor the NMDA receptors GluN2 and activate intracellular signaling complexes (Opazo et al., 2012). One study showed that Pin1 triggers structural changes in PSD-95 by interacting with PSD-95 at the T287, S290, and S295 consensus motifs and then negatively regulates its ability to interact with NMDARs (Antonelli et al., 2016). To be precise, Pin1 can bind with PSD-95 and then triggers its structural changes. Pin1-driven conformational rearrangements mainly impact on PDZ2, the domain involved in NMDAR recruitment. Larger NMDA-mediated synaptic currents in hippocampal slices were obtained from Pin1(–/–) mice compared with controls, and similar results were obtained in cultured hippocampal cells expressing a PSD-95 mutant unable to undergo prolyl-isomerization, further indicated the action of Pin1 on PSD-95 negatively regulates the ability of NMDARs. Furthermore, an increasing of dendritic spines was observed in Golgi-stained pyramidal neurons lacking Pin1 expression. Altogether, Pin1 could affect plasticity by determining the number of NMDARs initiating plasticity via PSD-95 prolyl-isomerization (Antonelli et al., 2016). Pin1 can also bind via its WW domain with phosphorylated threonine (T19) and serine (S25) in the N-terminal domain of PSD-95, and the binding reduces the palmitoylation of cysteine 3 and cysteine 5 in PSD-95 and then decreases its stability at excitatory synapses (Delgado, 2020; Delgado et al., 2020). This result indicates that Pin1 can modulate NMDARs via PSD-95 prolyl isomerization, suggesting that Pin1 plays a key role in synaptic transmission.

#### Alpha-amino-3-hydroxy-5-methyl-4-isoxazolepropionic acid receptors

AMPA receptors (AMPARs) are major regulators of excitatory synaptic transmission (Traynelis et al., 2010), and the expression of AMPARs at synapses is very dynamic (Shepherd and Huganir, 2007). AMPA receptors consist of four subunits (GluA1–GluA4), and any of them can form both homo and heteromers (Traynelis et al., 2010) and possess multiple phosphorylation sites (He et al., 2011). The phosphorylation of its subunits can regulate AMPAR function effectively (Kittler and Moss, 2006; Lee, 2006b), which is significant for modulating different forms of synaptic plasticity (Turrigiano and Nelson, 2004). Research has highlighted the role of the GluA1 subunit on serine 845 (S845) in various forms of synaptic plasticity (Lee, 2006a). The phosphorylation of GluA1-S845 is a necessary prerequisite step for *in vivo* sensory experience-dependent homeostatic synaptic plasticity of visual cortical neurons (Goel et al., 2011). GluA1-S845 phosphorylation is critical for maintaining the perisynaptic population of AMPARs by trafficking AMPARs to extrasynaptic sites and subsequent delivery to synapses during LTP (Oh et al., 2006). In addition, the phosphorylation of Ser831 and Ser845 are also considered key factors in LTP (Shepherd and Huganir, 2007). Additional studies have shown the potential utility of AMPA receptors as targets for seizure protection and validated AMPA receptors as novel targets for epilepsy therapy (Rogawski and Donevan, 1999; French et al., 2012, 2013; Krauss et al., 2012; Rogawski, 2013).

Recent studies have indicated that Pin1 may negatively regulate AMPA receptors. Pin1 KO upregulates epileptic susceptibility in mice, which is associated with an increase in AMPA receptors (Hou et al., 2021). The specific mechanism may involve calcium/calmodulin-dependent protein kinase II (CaMKII), protein kinase A (PKA) and protein kinase C isoenzymes (PKCs), the phosphorylation of which can effectively alter the excitatory function of AMPA receptors and provides a novel mechanism for channel modulation through a variety of protein signaling cascades (Jenkins and Traynelis, 2012; Wang et al., 2013).

CaMKII is a key mediator of AMPA receptors through its GluA1 subunit (Hayashi et al., 2000) and plays a critical role in the process of epilepsy. The phosphorylation of GluA1 at Ser831 via CaMKII could increase the conductance of AMPA receptors (Derkach et al., 1999; Kristensen et al., 2011). The activity of CaMKII is modulated by its regulatory domains. The regulatory domain blocks the substrate binding site of the CaMKII kinase domain in its basal state structures (Krapivinsky et al., 2004; Rellos et al., 2010; Chao et al., 2011; Coultrap and Bayer, 2012), while Ca^2+^/calmodulin can bind to the regulatory domain and then autophosphorylate CaMKII at Thr286 to keep the catalyst active (Partida et al., 2018). Recent studies using pull-down and immunoprecipitation assays have identified that Pin1 binds CaMKII in a phosphorylation-specific manner, which indicates the role of Pin1 in the phosphorylation-dependent signals involving CaMKII (Tatara et al., 2010). Additional research has demonstrated that the activity of CaMKII is significantly increased in Pin1 KO mice, and Pin1 can bind to phosphorylated CaMKII and decrease its activity (Shimizu et al., 2018; Hou et al., 2021). Moreover, restoration of the expression of Pin1 can reduce the phosphorylation of CaMKII and GluA1 (Hou et al., 2021), and Pin1 may regulate the phosphorylation of GluA1 Ser831 by acting on CaMKII, which supports the view that Pin1 effectively controls seizure susceptibility via the Pin1-CaMKII-AMPA receptor pathway.

Protein kinase C acts on a multitude of signal transduction pathways (Newton, 2001; Steinberg, 2008; Yang and Igumenova, 2013), and the relationship between PKC and AMPA receptors is significant in excitatory transmission. Some studies observed an increasing activity of PKC in the hippocampus of amygdala-kindled and hippocampus-kindled rats (Akiyama et al., 1995). PKC expression has also been detected to be decreased in pilocarpine induced epileptic rats, and further research has demonstrated that PKC can phosphorylate the GluA1 subunit of AMPA receptors at its S831 site (Roche et al., 1996; Kim et al., 2019). In addition, the C-terminal V5 domain is one of the most variable domains in PKCs due to cis-trans isomerization of the peptidyl-prolyl bonds (Yang and Igumenova, 2013). It has been shown that Pin1 binds to the hydrophobic motif of V5 and isomerizes the phosphorylated turn motif of conventional PKC, which primes it for subsequent ubiquitin-mediated degradation (Abrahamsen et al., 2012; Yang and Igumenova, 2013), to be specific, the study demonstrated that Pin1 could down regulate the conventional isozymes of PKC family. These studies highlight the role of the V5a-Pin1 interaction in neural function. Although PKC showed no change in Pin1 KO mice (Hou et al., 2021), studies have shown the effect of Pin1 on the PKCα subunit (Abrahamsen et al., 2012). Thus, the interaction between PKC and Pin1 and its function and molecular mechanism in the development of epilepsy is urgently needed.

Protein kinase A is crucial for both postsynaptic and presynaptic mechanisms underlying LTD and LTP (Kameyama et al., 1998; Banke et al., 2000), which supports its role in epilepsy. Previous studies have demonstrated that PKA can enhance neuronal AMPA receptor equilibrium response amplitude and upregulate channel opening frequency (Knapp et al., 1990; Greengard et al., 1991; Banke et al., 2000). Moreover, phosphorylation of AMPA is regulated by PKA, which increases the peak response open probability of the AMPA receptor by phosphorylating GluA1 Ser845 (Banke et al., 2000; Kim et al., 2019). In addition, the ratios of the expression of pPKA and pERK1/2 are decreased in epileptic rats, which further supports the phosphorylation function of activated PKA in epileptic rats (Kim et al., 2019). In terms of the relationship between PKA and Pin1, recent studies have supported the idea that PKA can modulate the phosphorylation of the WW domain of Pin1 at Ser16. The phosphorylation would reduce conformational heterogeneity and flexibility of the phospho-binding loop upon S16 phosphorylation and abrogates the binding capacity of Pin1 afterward, which would definitely affect the neuronal signaling in epilepsy (Luh et al., 2013; Smet-Nocca et al., 2013). Although there was no change in PKA in Pin1 knockout mice (Hou et al., 2021), the underlying mechanism of mutual regulation between Pin1 and PKA is still worth further study.

#### Gamma-aminobutyric acid receptors and glycine receptors

As previously discussed, synaptic inhibition is essential for shaping the dynamics of neuronal networks, and abnormal inhibition is closely associated with epilepsy (Macha et al., 2022). GABA receptors represent the most significant inhibitory system in the CNS (Barki and Xue, 2021). The receptors can be categorized as GABAA and GABAB. GABAA can modulate ligand-gated (or receptor-operated) ion channels, and GABAB can operate through second messengers. Both contribute to seizure-like discharge control when repetitive electrical stimulation is delivered to the limbic structure (Avoli and Lévesque, 2022). It has also been demonstrated that aberrant alternative GPHN splicing, leading to curtailed protein and diminished GABA receptors, is related to temporal lope lobe epilepsy (Förstera et al., 2010). Thus, GABAergic signaling is closely linked to the onset of epilepsy.

The glycine receptor (GlyR) is closely related to GABAA in the ‘gene superfamily’ of ligand-gated ion channels. It plays an essential role in mediating inhibitory neurotransmission in the spinal cord and brain stem, yet it also works as a coagonist with NMDARs in the CNS (Zafra et al., 2017). The malfunctions of GlyR are linked to temporal lobe epilepsy (Lynch et al., 2017), and it may contribute to the neuropsychiatric symptoms of the disease in a neuron type-specific way (Kankowski et al., 2017). Neuron type-specific expression of a gain-of-function variant of GlyR has been observed in the hippocampus of patients with temporal lobe epilepsy (Winkelmann et al., 2014). In addition, preclinical studies have shown that inhibition of its substrate selective transporter, glycine transporter-1 (GlyT-1), can promote neuroprotection and provide a pharmacotherapeutic strategy for epilepsy (Cioffi and Guzzo, 2016). These studies support the link between GlyR and epilepsy.

Accumulating evidence has demonstrated the interaction of Pin1 with GABA receptors and GlyR. It is known that Gephyrin is a central component of GABAergic signaling and plays a key role in α2 and γ2 subunit-containing GABAAR clustering (Kneussel et al., 1999; Tretter et al., 2012; Antonelli et al., 2014). The NL2 isoform is a kind of adhesion molecule that is present in GABAergic PSDs (Varoqueaux et al., 2004). The interaction of NL2 and gephyrin can modulate NL-scaffolding protein interactions and regulate excitatory and inhibitory synaptic transmission (Antonelli et al., 2014). Pin1 has been shown to interact with gephyrin and alter its overall structure, thereby enhancing its ability to bind GlyR (Zita et al., 2007; Antonelli et al., 2014). Additionally, Pin1 can mediate propyl isomerization of phosphorylated serine 714. Further studies have shown that Pin1 negatively regulates the interaction of the gephyrin–NL2 complex formation, which in turn downregulates GABAergic synaptic transmission (Antonelli et al., 2014), suggesting the existence of a Pin1/NL2/gephyrin signaling pathway in the regulation of GABAergic synapses. Moreover, ten putative Pin1 consensus motifs have been identified, mostly concentrated in the C-domain of gephyrin. The C-domain’s cluster was responsible for Pin1 recruitment, following by Pin1-driven conformational changes of gephyrin substrate. Such structural remodeling of gephyrin could affect its binding affinity for the β subunit of the GlyR without affecting its oligomerization properties. Consistent with these findings, hippocampal neurons derived from Pin1 knockout mice show a loss in the number of GlyR immunoreactive puncta, corresponding to a reduction in amplitude of glycine-induced current (Zacchi et al., 2014). These studies further demonstrate the mechanism by which Pin1 regulates GlyR function.

#### Ion channels

Ion channels also participate in the occurrence of epilepsy. Approximately 25% of genes identified in epilepsy encode ion channels (Oyrer et al., 2018), and electrophysiological studies have shown not only pathophysiological mechanisms underlying epilepsy but also the mechanism of action of several antiepileptic drugs involve ion channels (Sugiura and Ugawa, 2017). Research has shown that when AMPA receptors are activated, they allow Na^+^ to enter or leave, and extracellular Na ^+^ influx causes postsynaptic membrane depolarization and induces EPSP to participate in excitatory synaptic transmission. At the same time, membrane depolarization induced by the AMPA receptor causes Mg^2+^ to move away from the NMDA receptor channel and open the NMDA receptor channel. Activated NMDA receptors result in excessive Ca^2+^ influx and intracellular calcium overload, leading to cell death. In addition, activated GABA receptors can induce the opening of chloride ion channels, and the rapid influx of Cl^–^ causes hyperpolarization of the postsynaptic membrane and generates inhibitory postsynaptic potentials that inhibit neuronal excitation (Lippman-Bell et al., 2016).

In terms of the relationship between Pin1 and ion channels, a Pin1-dependent mechanism has been reported to regulate the association of the A-type K^+^ channel subunit Kv4.2, suggesting a Pin1-mediated mechanism in the regulation of reversal learning (Hu J. H. et al., 2020). Recent studies have also hypothesized that hypoxia-induced changes in the flavoprotein (Fp)-mediated redox ratio of the carotid body (CB) modulate the Pin1/p47phox tandem to alter type I cell potassium channels and therefore chemoreceptor discharge (CND) (Bernardini et al., 2020). Pin1 also regulates Ca^2+^ influx by binding to salt-induced kinase 2 (SIK2) and decreasing the expression of p35, a negative regulator of Ca^2+^ influx (Nakatsu et al., 2017), yet the effect is induced by high glucose, which is not the same as the microenvironment of epilepsy. The association of Na(+)/H(+) exchanger regulatory factor (NHERF)-1 with Pin1 may also indicate the role of Pin1 in the Na(+)/H(+) channel (He et al., 2001). The mechanism by which Pin1 regulates epileptic ion channels may illuminate the underlying roles of Pin1 in epilepsy.

### Pin 1 in neuronal signaling associated with epilepsy

#### Notch1 signaling pathway

Notch1 is a membrane receptor in the Notch family (Arumugam et al., 2018). Notch1 is tightly connected to cell proliferation, differentiation, and developmental fate switching (Arumugam et al., 2018). For neurons, Notch1 plays various roles in the development of the CNS, participating in the proliferation, survival, self-renewal and differentiation of neural stem cells (NSCs) (Lathia et al., 2008; Arumugam et al., 2018). In addition, Notch1 signaling may also affect synaptic plasticity and learning and memory in the adult brain (Lathia et al., 2008; Alberi et al., 2013; Sargin et al., 2013; Arumugam et al., 2018). In terms of epilepsy, Notch1 signaling has the ability to induce astrogliosis in glioma, which is critical in the occurrence of epilepsy. Notch signaling has been observed to be downregulated in a status epilepticus model, and the decrease may be associated with the disruption of the stem cell niche (Sibbe et al., 2012). Another study suggested that the Notch1 signaling pathway is activated in TLE mouse models and that its function is closely related to microglial phenotypic transformation (Deng et al., 2020). In addition, Notch1 was observed to increase in rats with status epilepticus and spontaneous recurrent seizures (Liu et al., 2014). Overall, the Notch1 signaling pathway is significant in the pathogenesis of epilepsy. Notch1 was found to modulate hippocampal plasticity via interaction with the reelin pathway, glutamatergic transmission and CREB signaling (Brai et al., 2015). CaMKII is closely related to Notch signaling activation by accelerating the degradation of the silencing mediator of retinoic acid and thyroid hormone receptor (SMRT), a corepressor of Notch signaling (Ann et al., 2012). In addition, the coaction of the PKA pathway and Notch1 expression is essential for the differentiation of C6 astrocytes (Angulo-Rojo et al., 2013). These studies highlight the interaction of CaMKII and PKA with the Notch1 signaling pathway.

Furthermore, Pin1 interacts with the Notch1 pathway. A strong correlation between Pin1 overexpression and high levels of activated Notch1 has been observed in breast cancer patients, and some studies have suggested that Notch1 may directly induce transcription of Pin1 (Rustighi et al., 2009). Resveratrol downregulates Pin1 and Notch1 intracellular domain (NICD1) at the same time and reverses cell dysfunction by inhibiting the Pin1/Notch1 signaling pathway (Yu and Fang, 2022). The relationship between NIC and Pin1 has also been analyzed in Pin1/p53 double-knockout mice, Pin1 deficiency might affect NIC stabilization, which is associated with the activity of presenilin1, a component of the gsecretase complex that would affect the releasing of NIC (Takahashi et al., 2008). Pin1 can also mediate cell death through the p53-NICD complex in ischemic stroke (Arumugam et al., 2018). The results indicated that the Pin1-p53-NIC system might be essential for preventing the pathogenesis and progression of epilepsy. Further research has demonstrated that Pin1 could bind with the crucial phosphorylated sites within the Ser-Thr-rich (STR) region of Notch1, then the conformational changes would lead to cleavage by γ-secretase and increased release of the active intracellular domain of Notch1, which explain the role of Pin1 in Notch1 pathway (Rustighi et al., 2009). Pin1 may promote proapoptotic function in neuronal death by promoting NICD1 stability (Baik et al., 2015), while other argue that Notch1 may increase the expression level of anti-apoptotic gene in rats with cerebral infarction and act an anti-apoptotic function in neuron (Zhao et al., 2019). Besides, Pin1 is closely associated with anti-apoptosis protein BCL-2 (Sultana et al., 2006; Li et al., 2007) and depict an anti-apoptotic effect in AD neurons (LaFerla et al., 1996; Rong et al., 2017; Wang et al., 2023). These findings strongly point to an anti-epileptic effect of Pin1 by antagonizing neuronal apoptosis in epilepsy, thus Pin1-Nocth1 complex may play anti-epileptic role.

**FIGURE 2:**
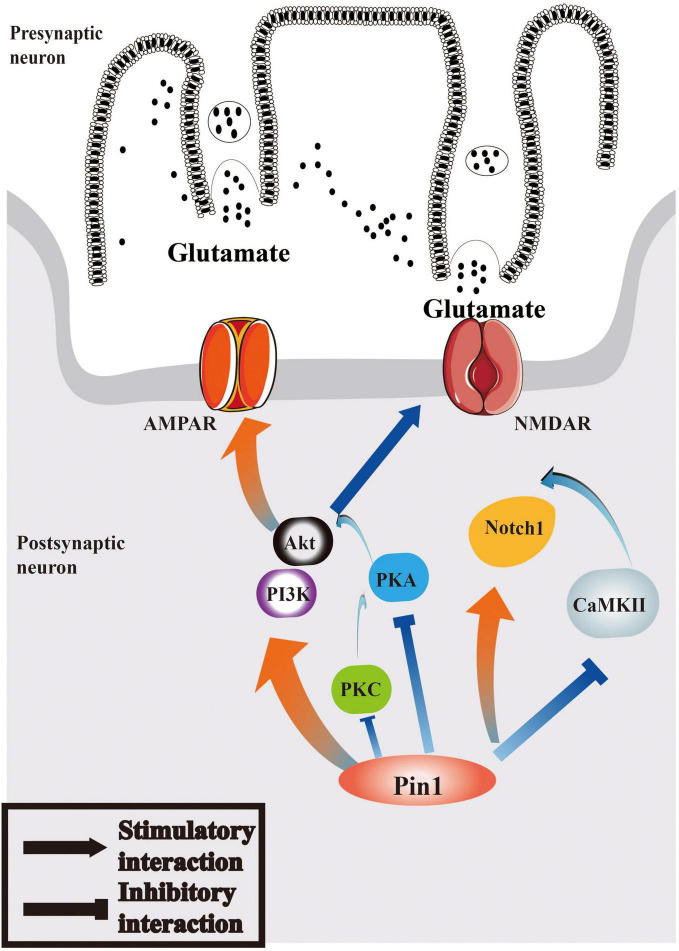
Pin1 may regulate Notch1 signaling and PI3K/Akt signaling pathways in epilepsy. Pin1 has an interaction with Notch1 pathways, and the function may associated with CaMKII. AMPAR and NMDAR can both be mediated by PI3K/Akt pathway, whose activation will be regulated by PKA, PKC, and Pin1. The relationship between Pin1 and PKA, PKC and CaMKII deserved further research.

Therefore, we can hypothesize that the interaction between Pin1 and Notch1 pathway may play a role in epilepsy and that Notch1 may participate in epileptogenesis via the function of CaMKII and PKA. The relevant characteristics of the Notch1 pathway are summarized in Figure 2.

#### PI3K/Akt signaling pathway

The PI3K/Akt pathway is significant for growth and survival and participates in many biological processes. It has been demonstrated that the PI3K/Akt pathway is associated with early ischemia/reperfusion injury (Fu et al., 2014), and it can regulate neuronal apoptosis and neuroinflammation in Alzheimer’s disease (Liu and Li, 2020). Recent studies have shown that PI3K/Akt signaling improves the survival of neurons and axonal growth as well (Huang et al., 2017). For epilepsy, the PI3K/Akt signaling pathway can effectively inhibit the occurrence of epilepsy by attenuating neuronal apoptosis and autophagy (Duan et al., 2018; Wei et al., 2018; Hu F. et al., 2020). Moreover, the PI3K/Akt signaling pathway synergistically enhances adipogenesis and glucose uptake in porcine bone marrow mesenchymal stem cells (pBMSCs) through CaMKII (Zhang et al., 2018). Although few data describe the interaction between CaMKII and the PI3K/Akt pathway in neurons, it is logical to infer their role in epilepsy. In addition, fluoxetine elicited neuroprotection via PKA and PI3K/Akt (Zeng et al., 2016), which suggests the interaction of the PI3K/Akt signaling pathway and PKA in neurons. Furthermore, PKA-PI3K-Akt signaling has the ability to modulate the AMPA receptor GluA1 subunit to assist in learning and memory. These data demonstrate the coaction of PKA and PI3K/AKT and demonstrate their function on the AMPA receptor, which has a critical role in the progression of epilepsy (Chen M. W. et al., 2020). The relationship between PKC and PI3K/AKT has also been studied. PKC can regulate the activation of PKA-PI3K-Akt signaling, which effectively broadens the knowledge of the downstream signaling of GluA1 (Chen M. W. et al., 2020). Recent studies have also shown that the PI3K/Akt pathway may affect NMDA receptors via TrKB and BDNF (Srivastava et al., 2018), and activation of the PI3K-AKT-mTOR pathway can block NMDA-induced autophagy in neurons (Wang et al., 2014). The studies above have identified the interaction of PI3K/AKT and synaptic receptors and highlighted the effects of the PI3K/AKT pathway in epilepsy.

Pin1 can facilitate the activation of PI3K/Akt signaling. Some research has suggested that the expression of Pin1 is positively associated with activation of PI3K/Akt signaling (Yang et al., 2020), and that Pin1 can phosphorylate Akt at Ser434 and then increase Akt stability and subsequently activate PI3K/Akt (Nakatsu et al., 2011). The deletion of Pin1 can block the function of PI3K/Akt and suppress the growth of cells (Zhang et al., 2019). However, some studies have indicated an inhibitory effect of Pin1 on the PI3K/Akt pathway. Pin1 may downregulate the action of the PI3K/Akt/mTOR pathway and play a protective role in senescent cells (Zhang et al., 2021). The detailed mechanisms by which Pin1 acts on PI3K/Akt signaling may be useful to explain the role of Pin1 in neurons. Notably, Pin1 enables phosphatase PP2A to further mediate tau dephosphorylation in the AD brain (Ramakrishnan et al., 2003). PP2A is a special protein phosphatase that can dephosphorylate Akt. This function further supports the role of the Pin1/PI3K/Akt interaction in neurons. Therefore, it is valuable to study the mechanism by which Pin1 acts on the PI3K/Akt pathway, and PI3K/Akt may be another potential target for epilepsy treatment. The function of the PI3K/Akt pathway is shown in Figure 2.

### Pin 1 and tau in epilepsy

Tau is an intrinsically disordered protein which has a closely relationship with microtubules. Its interaction with microtubules is characterized by a rapid “kiss-and-hop” process. It is most abundant in neuronal axons and the rapid interaction may explain how tau binding to microtubules without disturb axonal transport (Janning et al., 2014; Wang and Mandelkow, 2016; Chang et al., 2021b). Tau is also present in neuronal cell bodies, nuclei, synaptic specializations, glia and other cell types in the brain and peripheral organs (Wang and Mandelkow, 2016; Ittner and Ittner, 2018), which indicated that it may be involved in a variety of common and disabling brain diseases, including epilepsy. Reducing levels of endogenous tau has protective effects in epilepsy experimental models (DeVos et al., 2013; Gheyara et al., 2014). Tau deficit mice have an increased frequency of inhibitory postsynaptic currents in dentate granule cells (Roberson Halabisky et al., 2011), which may explain the beneficial effects of decreasing tau in epilepsy. Further study observed that Tau ablation could increase the excitability of inhibitory neurons, and structurally alters their axon initial segments, thus promote inhibition and suppress hypersynchrony (Chang et al., 2021a). Together, tau reduction prevents the occurrence of epilepsy. Hyperphosphorylated Tau protein might contribute to vulnerability to epileptogenesis, for excessive post-translational modifications of Tau creates a pathologic environment promoting epileptogenesis (Paudel et al., 2019). Therefore, therapy targeting Tau protein and p-tau anti-may be a viable epileptogenic strategy.

The interaction between Pin1 and tau has been demonstrated. Pin1 binds to pT231-tau and promotes tau dephosphorylation, and further study confirmed that the binding of Pin1 and p-tau could in turn isomerize phosphorylated tau proteins so that their conformation changes from *cis* to *trans*, thus favoring its recognition by the phosphatase PP2A. PP2A has conformational specificity and dephosphorylates only the trans pS/T-P motif (Zhou et al., 2000; Luna-Muñoz et al., 2007; Wang et al., 2020). *Cis* pT231-tau is associated with neuronal apoptosis under neuronal stress, thus is a key role in neurodegeneration, while Pin1 has the ability to decrease the level of abnormal p-tau and the regulation is a critical mechanism to protect against tau-related pathology (Wang et al., 2020).

### Pin1 and the therapeutic application of epilepsy

The onset of seizure is mainly due to sudden abnormal discharge of neurons in the brain leading to temporary brain dysfunction. Thus, controlling or significantly reducing the abnormal charging of neurons is the main treatment for epilepsy. As mentioned above, Pin1 plays a critical role in preventing pathologies in epilepsy by regulating synapses; thus, it is reasonable to speculate that the upregulation and/or activation of Pin1 may be helpful for epilepsy treatment. However, Pin1 is a well-known oncogene, and overexpression of Pin1 has been reported to be associated with several cancers (Zhou and Lu, 2016). It can effectively activate a number of oncogenes and inactivate various tumor suppressors by modulating their functions to promote the development of cancer (Cheng and Tse, 2019; Yu et al., 2020). Therefore, treatment with Pin1 should be carried out with caution. Delivering the Pin1 activator to neurons specifically or therapies targeting the upstream regulators of Pin1 may be optional therapeutic strategies (Wang et al., 2020). In addition, although no drug candidates targeting Pin1 for neuronal diseases have been reported, specific antibodies targeting Thr231 of tau, which is the Pin1 binding site, have been developed, and such monoclonal antibodies could effectively prevent tau-related pathology development in AD and TBI patients by entering neurons and blocking the induction of pathological *cis* p-tau (Shahpasand et al., 2018; Chen D. et al., 2020; Wang et al., 2020), which is closely related to cognitive impairment and dementia (Albayram et al., 2017; Qiu et al., 2021). Moreover, aberrant p-tau aggregation has been reported in the epileptic human brain and in animal models of epilepsy (Machado et al., 2019; Liu et al., 2021; Canet et al., 2022), highlighting the role of pathological p-tau in the onset of epilepsy, suggesting that a therapy targeting the Pin1 binding site, especially p-tau, may also be a potential anti-epileptogenic therapy (Liu et al., 2016). Brivaracetam and the non-competitive antagonist of the AMPA glutamate receptor perampanel are the more recent antiseizure medications with a low risk of interaction, and have recently been approved for monotherapy and/or adjunctive treatment in the USA and Europe (Rohracher et al., 2021). Because of the close relationship between the AMPA receptor and Pin1, this novel therapy may broaden the spectrum of Pin1 in the treatment of epilepsy. More research in animal models and technological innovation would be helpful to further boost the success of Pin1 in epilepsy treatment.

## Conclusion

The evidence discussed in this review supports the idea that Pin1 is implicated in the onset and progression of epilepsy. Pin1 is identified as a unique enzyme and a crucial regulator that precisely regulates the *cis-trans* isomerization of a wide variety of phosphorylated proteins and alters the activity of its target proteins. Regulation of the phosphorylation and transcriptional landscapes by Pin1 makes this enzyme a central effector of many physiological and pathological activities related to neuronal cells (Zannini et al., 2019). In line with this hypothesis, Pin1 has been reported to take part in neuro-associated diseases (Fagiani et al., 2021), including epilepsy. In addition, emerging evidence supports the notion that Pin1 is a key molecule in epileptogenesis. Pin1 has strong neuroprotective effects in the progression of epilepsy, and the Pin1/NMDAR complex, Pin1-CaMKII-AMPA receptor axis and Pin1-NL2/gephyrin- GABAergic signaling may all be involved in the mechanism (Antonelli et al., 2014, 2016; Tang et al., 2017; Hou et al., 2021). The data strongly suggest the potential utility of Pin1 as a target for seizure protection (Hou et al., 2021). The function of Pin1 in epilepsy may be associated with some classical signaling pathways, such as the Notch1 signaling and PI3K/Akt signaling pathways. However, more evidence is needed to clarify the potential role of Pin1 in neuronal signaling, particularly in the occurrence of epilepsy. Finally, Pin1-related treatment is promising, and more research and technological innovation are needed to overcome its limitations in direct administration. We anticipate that the specific molecular mechanisms will be elucidated in the coming years, pushing Pin1 toward true clinical application.

## Author contributions

All authors: conceptualization, substantive supervision, revision over the article, read and agreed to the published version of the manuscript. YC: collection the literature and writing—review and editing the manuscript. HL: project administration and funding acquisition.

## Abbreviations

HFOs: high-frequency oscillations
rHFOSs: repetitive HFOs and spikes
PPIases: the peptidyl prolyl cis-trans isomerases
PPase: protein phosphatase
NIMA: never in mitosis, gene A
APP: amyloid precursor protein
Pin1: PPIase NIMA-interacting 1
LTP: long-term potentiation
TLE: temporal lobe epilepsy
CNS: central nervous system
SH3: Src homology 3
PDZ: PSD-95/Disks large/zona occludens-1
CaMKII: calcium/calmodulin-dependent protein kinase II
PKA: protein kinase A
PKCs: protein kinase C isoenzymes
GlyT-1: glycine transporter-1
NL2: Neuroligin2
CB: carotid body
CND: chemoreceptor discharge
SIK2: salt-induced kinase 2
NHERF: Na(+)/H(+) exchanger regulatory factor
NSC: neural stem cell
SMRT: silencing mediator of retinoic acid and thyroid hormone receptor
NICD1: Notch1 intracellular domain
pBMSCs: porcine bone marrow mesenchymal stem cells.
